# Prognostic and Clinicopathological Value of Programmed Death Ligand-1 in Breast Cancer: A Meta-Analysis

**DOI:** 10.1371/journal.pone.0156323

**Published:** 2016-05-26

**Authors:** Yawen Guo, Pan Yu, Zeming Liu, Yusufu Maimaiti, Shan Wang, Xingjie Yin, Chunping Liu, Tao Huang

**Affiliations:** Department of Breast and Thyroid Surgery, Union Hospital, Tongji Medical College, Huazhong University of Science And Technology, Wuhan, China; Fondazione IRCCS Istituto Nazionale dei Tumori, ITALY

## Abstract

Recently, the interest in programmed death ligand-1 (PD-L1) as a prognostic marker in several types of malignant tumors has increased. In the present meta-analysis, we aimed to explore the prognostic and clinicopathological value of PD-L1 in breast cancer. We searched Medline/PubMed, Web of Science, EMBASE, the Cochrane Library databases, and grey literature from inception until January 20, 2016. Studies concerning breast cancer that focused on PD-L1 expression and studies reporting survival data were included; two authors independently performed the data extraction. The pooled risk ratio (RR) and 95% confidence interval (CI) were assessed to determine the association between the clinicopathological parameters of patients and PD-L1 expression. Five studies involving 2061 patients were included in this meta-analysis. The results indicated that positive/higher PD-L1 expression was a negative predictor for breast cancer, with an RR of 1.64 (95% CI, 1.14–2.34) for the total mortality risk and an RR of 2.53 (95% CI, 1.78–3.59) for the mortality risk 10 years after surgery. Moreover, positive/higher PD-L1 expression was significantly associated with positive lymph node metastasis (RR, 1.33; 95% CI, 1.04–1.70), poor nuclear grade (RR, 1.24; 95% CI, 1.07–1.43), and negative estrogen receptor status (RR, 2.45; 95% CI, 1.31–4.60) in breast cancer patients. Our findings suggest that PD-L1 can serve as a significant biomarker for poor prognosis and the adverse clinicopathologic features of breast cancer and could facilitate the better management of individual patients.

## Introduction

Breast cancer is currently the most frequently diagnosed cancer and is the leading cause of cancer-related death in women; in fact, in 2012, a total of 1.7 million new cases breast cancer and 521,000 cases of breast cancer-related mortality were reported worldwide [1]. At present, the prognosis, classification, and treatment of breast cancer is dependent on tumor histological grading, lymph node stage, and tumor stage (TNM), as well as 3 major protein markers: estrogen receptor (ER), progesterone receptor (PR), and human epidermal growth factor (EGF) receptor 2 (HER2) [2, 3]. However, breast cancer is generally not considered an immunogenic malignancy. Even though breast cancer cells use immune pathways to evade antitumor immune responses and progressively grow and metastasize, no treatment that enhances the antitumor immune response is currently used [4]. However, some investigators have begun to focus on novel immunotherapeutic strategies for treating breast cancer.

The expression of the programmed cell death 1 (PD-1), a member of the B7 family of immune-regulatory cell-surface proteins, and its cognate ligand PD-L1, within the tumor microenvironment is a major resistance mechanism for escaping immune surveillance [5, 6]. PD-L1 is expressed in tumor-infiltrating lymphocytes and tumor cells of cancer including breast, lung, prostate, gastrointestinal, and malignant melanomas [7–11]. Furthermore, PD-L1 expression is associated with poor prognosis in breast, pancreatic, and renal cell cancers [12–18]. To better understand the potential relationship between PD-L1 and prognosis in breast cancer, it should be clarified whether PD-L1 is a possible target for the treatment of breast cancer.

Although some studies have been conducted to analyze the relationship between PD-L1 and breast cancer, its prognostic role in breast cancer remains controversial. To our knowledge, no meta-analyses have been performed on this topic thus far. In this study, we aimed to perform an up-to-date meta-analysis to determine the prognostic value of PD-L1 in breast cancer.

## Methods

This study was conducted and reported according to the Preferred Reporting Items for Systematic Reviews and Meta-Analyses (PRISMA) statement checklist (S1 File).

### Data search strategy

We searched the Medline/PubMed, Web of Science, EMBASE, the Cochrane Library databases, and grey literature from inception January 20, 2016. The search strategy used both MeSH terms and free-text words to increase sensitivity. The key terms employed for literature retrieval included “PD-L1,” “programmed death ligand-1,” “CD274,” “B7-H1,” or “B7 homolog 1”; “breast cancer,” “breast carcinoma,” or “breast tumor”; and “survival,” “outcome,” or “prognosis. We also contacted the corresponding authors to obtain any additional information, if necessary.

### Inclusion and exclusion criteria

Articles were selected if they met the following criteria: (i) they were focused on breast cancer; (ii) all selected cancer patients were confirmed as having breast cancer via pathological examination; and (iii) the correlation between PD-L1, clinicopathological features, and prognosis was discussed. Studies were excluded if they met any of the following criteria: (i) duplicate publication; (ii) non-human experiments were performed, non-English papers; (iii) conference abstract; (iv) review articles, case reports, or letters; or (v) insufficient data regarding 95% confidence interval (95% CI) and risk ratios (RR) provided, or (vi) the Kaplan-Meier curve could not be extracted. In cases where more than one article was published from the same center, the study with the information most relevant to the present study was included.

### Data extraction

Two independent reviewers (Yawen Guo and Pan Yu) extracted all the data, and the following information was recorded: author, year of publication, patient number, country, specimen, detection method, cut-off values for the positive rates of PD-L1 overexpression, duration of follow-up after surgery, study end points, and data presented in the tables and figures. For articles that only provided survival data in a Kaplan Meier curve, the survival rates were calculated using Engauge Digitizer software, version 3.0 (http://digitizer.sourceforge.net) to reconstruct the RR estimate and its variance, assuming that patients were censored at a constant rate during follow-up. The quality of the selected articles was assessed according to the Newcastle Ottawa Scale [19].

### Statistical analysis

Statistical analysis was performed according to the guidelines proposed by the MetaAnalysis of Observational Studies in Epidemiology (MOOSE) group [20]. Data from each study were analyzed using Review Manager software, version 5.3 (Copenhagen: The Nordic Cochrane Centre, The Cochrane Collaboration, 2014) and Stata SE12.0 (Stata Corporation, TX, USA). Funnel plots were used to assess publication bias, and p values of <0.05 were considered statistically significant. An RR of >1 indicated worse survival for patients with high PD-L1 expression, whereas an RR of <1 implied a survival benefit. The RRs and their 95% CIs were used to assess the correlations between PD-L1 expression and the clinicopathological features of breast cancer, including tumor size; TNM stage; nuclear grade; lymph node metastasis; and the expression of ER, PR, Her-2, and Ki67. The pooled RR of each study was calculated using a fixed-effects model if there was no significant heterogeneity occurred among the studies, whereas a random-effects model was adopted if heterogeneity was observed. The heterogeneity among the data was evaluated by using the chi-square test and the I^2^ statistic. An I^2^ value of >50% of the I^2^ statistic was considered to indicate significant heterogeneity [21]. All the p values were two-tailed.

## Results

### Search results

**Fig 1:**
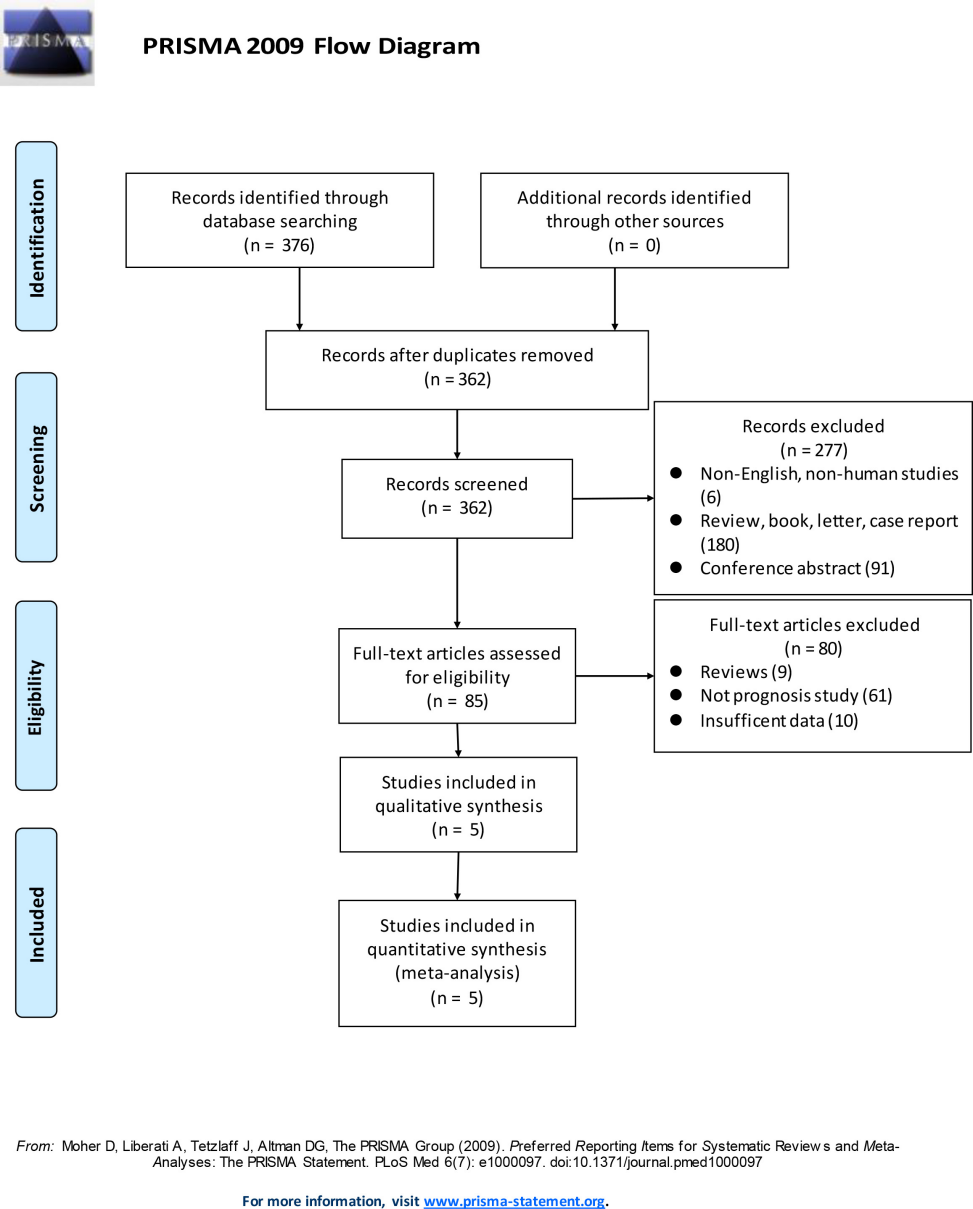
Flowchart of the study selection process.

We identified 376 articles using our search strategy (S2 File). After screening the titles and abstracts, we excluded 291 articles because they were not original articles (e.g., review, letter, case report), not breast cancer-related studies, not English language papers, not human studies, or were conference abstracts. After reviewing the complete text of 85 articles, 80 articles were excluded because some of them were review articles, case reports, or letters; while some articles did not have sufficient data regarding the 95% confidence interval (CI) and risk ratios (RRs), or the Kaplan Meier data could not be extracted, leaving only 5 studies involving 2061 patients that were included in the meta-analysis. The details of the screening procedure are illustrated in Fig 1. All the enrolled articles comprehensively assessed the expression of PD-L1, clinicopathological features of breast cancer, and survival rate.

### Study selection and characteristics

The details of the 5 eligible studies are listed in Table 1 [13–17]. The publication years of the eligible studies ranged from 2014 to 2016, and the number of patients in each study ranged from 36 to 870. The PD-L1 expression levels were measured in tumor tissues in all 5 studies, including 4 studies wherein tissue immunochemical staining (IHC) was used to detect PD-L1 expression and 1 study wherein DNA contents (determined by flow cytometry) were used to survey the genomes of each tumor. As indicated in Table 1, each article had a specific cut-off value, which consequently influenced the positive rates of PD-L1 overexpression in each study. Moreover, the mean duration of follow-up after surgery ranged from 4.7 to 9.8 years. The Disease-free survival (DFS) and overall survival (OS) were used as end points in 4 studies, whereas OS alone was used in 1 study as the end point (Table 1). Furthermore, the clinicopathological features including tumor size; TNM stage; nuclear grade; lymph node metastasis; and expression of ER, PR, Her-2, and Ki67 were reported in all 5 studies.

**Table 1:** Main characteristics of the studies included in this meta-analysis.

First author of study	Year	Number of patients	Country	Specimen	Detection method	Cut-off (positive/High expression)	Follow up (years)	End point
Muenst	2014	650	Switzerland	Tissue	IHC	H-Score ≥ 100 (23.4%)	5.4 (0.08–14.5)	OS
Barrett	2015	36	America	Tissue	DNA content Flow cytometry	High level (log2 ratio ≥ 1) amplicon (22.2%)	4.7 (0.9–12.0)	DFS/OS
Park	2015	316	Korea	Tissue	IHC	H-Score ≥ 3 + (51.6%)	9.8 (0.4–12.8)	DFS/OS
Qin	2015	870	China	Tissue	IHC	≥5% tumor cell staining (21.7%)	8.2 (1.4–22.1)	DFS/OS
Baptista	2016	189	Brazil	Tissue	IHC	Median (56.6%)	7.18^1^	DFS/OS

^1^median

DFS, disease-free survival; H-score, Histo-score; OS, overall survival

### Main results

**Fig 2:**
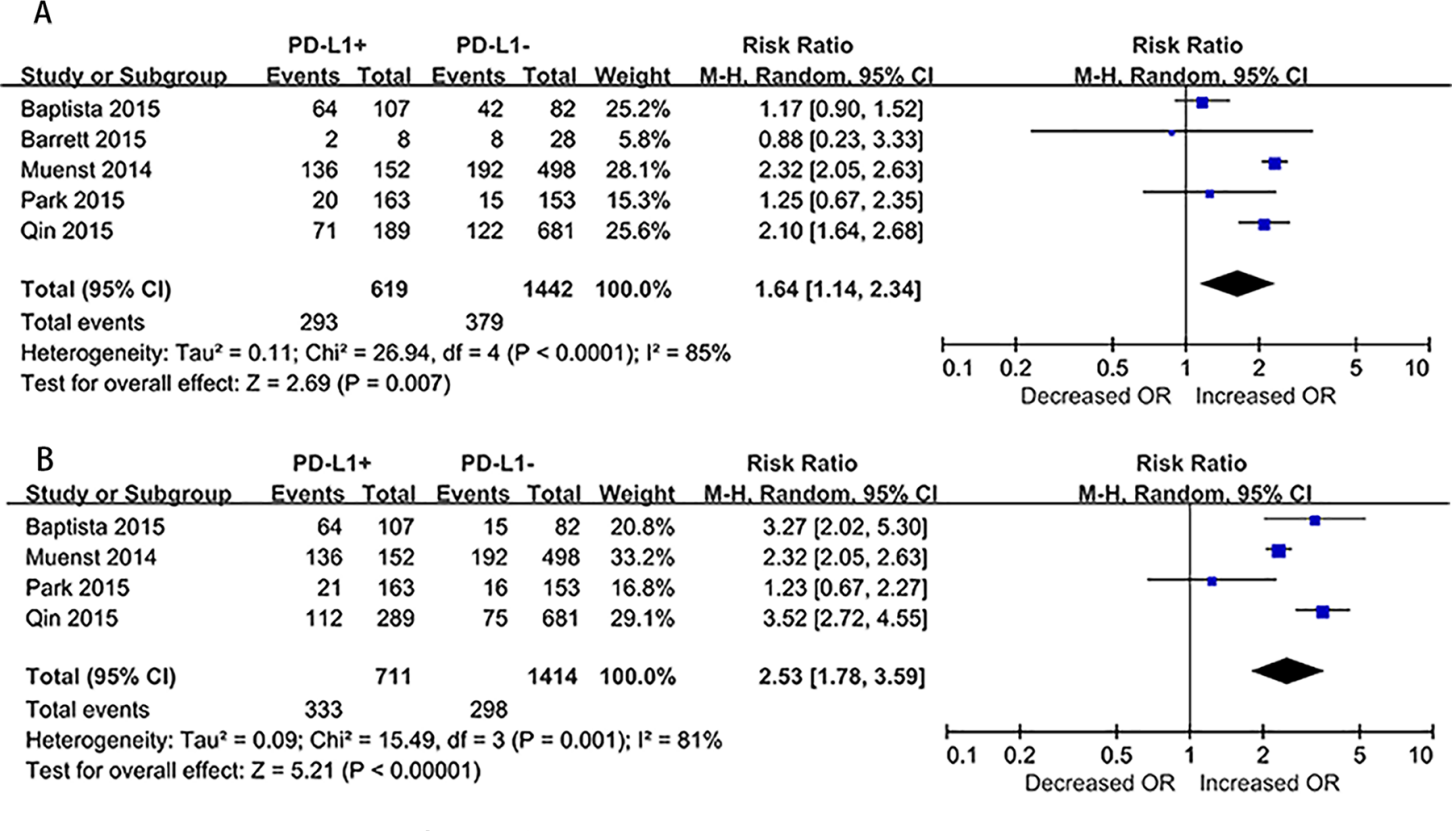
Forest plots of studies evaluating risk ratios (RRs) of PD-L1 for breast cancer specific survival. (A) Total mortality risk (MR) among breast cancer patients. (B) The MR 10 years after surgery (MR_10years_) in breast cancer patients.

As noted in Fig 2, positive PD-L1 expression significantly associated with enhanced total mortality risk (MR) among breast cancer patients in the random-effects model; the pooled RR was 1.64 (95% CI, 1.14–2.34), despite the presence of heterogeneity among the studies (I² = 85%, p < 0.0001; Fig 2A). Due to differences in the follow-up duration of each study, we assessed the mortality risk 10 years after surgery (MR_10years_) using a random-effects model and data from 4 articles with sufficient data. The RR was 2.53 (95% CI, 1.78–3.59), despite the presence of significant heterogeneity among the studies (I^2^ = 81%, p = 0.001; Fig 2B). Due to the presence of significant heterogeneity in the mortality risk across the studies, we further examined the potential sources of heterogeneity through metaregression, and found that the year of publication, detection method, and analysis method did not contribute to the heterogeneity.

**Fig 3:**
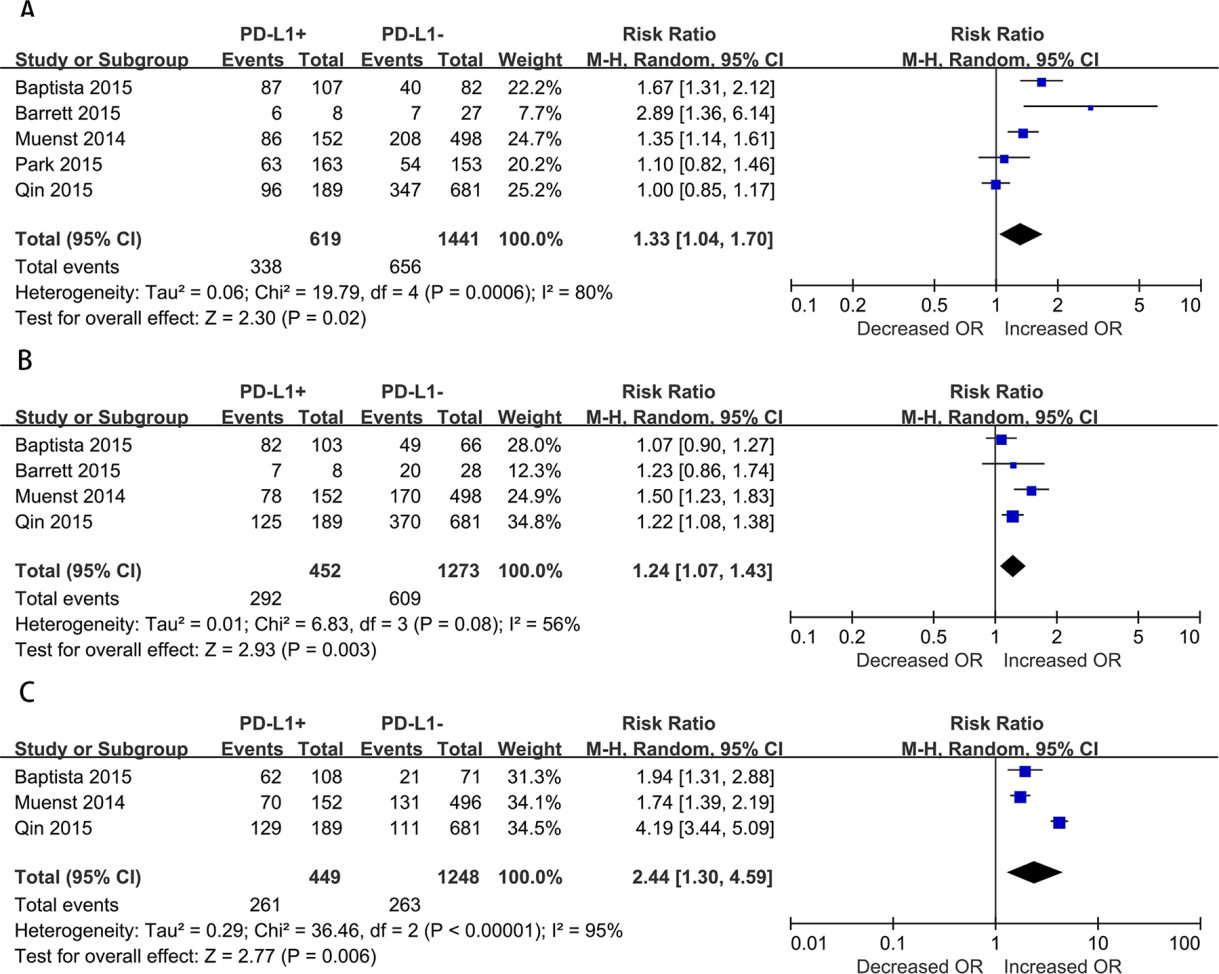
Forest plots of studies evaluating the association between PD-L1 and clinical parameters in breast cancer. (A) Lymph node metastasis (positive versus negative). (B) Nuclear grade (3, 4 versus 1, 2). (C) ER status (negative versus positive).

Moreover, we assessed the relationship between positive PD-L1 expression and clinicopathological features (Fig 3). Lymph node metastasis of breast cancer was reported in all 5 studies. Due to the presence of significant heterogeneity (I^2^ = 80%, p = 0.0006), the random-effect model was adopted, which indicated a pooled RR (PD-L1-positive versus PD-L1-negative) of 1.33 (95% CI, 1.04–1.70; Fig 3A). In addition, 4 studies reported on the nuclear grade of breast cancer. Due to the presence of significant heterogeneity (I^2^ = 56%, p = 0.08), a random-effects model was adopted, which indicated a pooled RR (3 versus ≤2) of 1.24 (95% CI, 1.07–1.43; Fig 3B). Furthermore, 3 studies reported on the ER status of breast cancer. Here as well, due to the presence of significant heterogeneity (I^2^ = 94%, p < 0.00001), the random-effect model was adopted, which indicated a pooled RR (PD-L1-negative versus PD-L1-positive) of 2.45 (95% CI, 1.31–4.60; Fig 3C). However, no significant relationship was observed between PD-L1 overexpression and other clinical characteristics such as tumor size; TNM stages; and expression of PR, Ki67, and Her2 in breast cancer due to insufficient data. Furthermore, high PD-L1 expression was significantly associated with lymph node metastasis, poor nuclear grade, and negative ER expression.

### Publication bias

**Fig 4:**
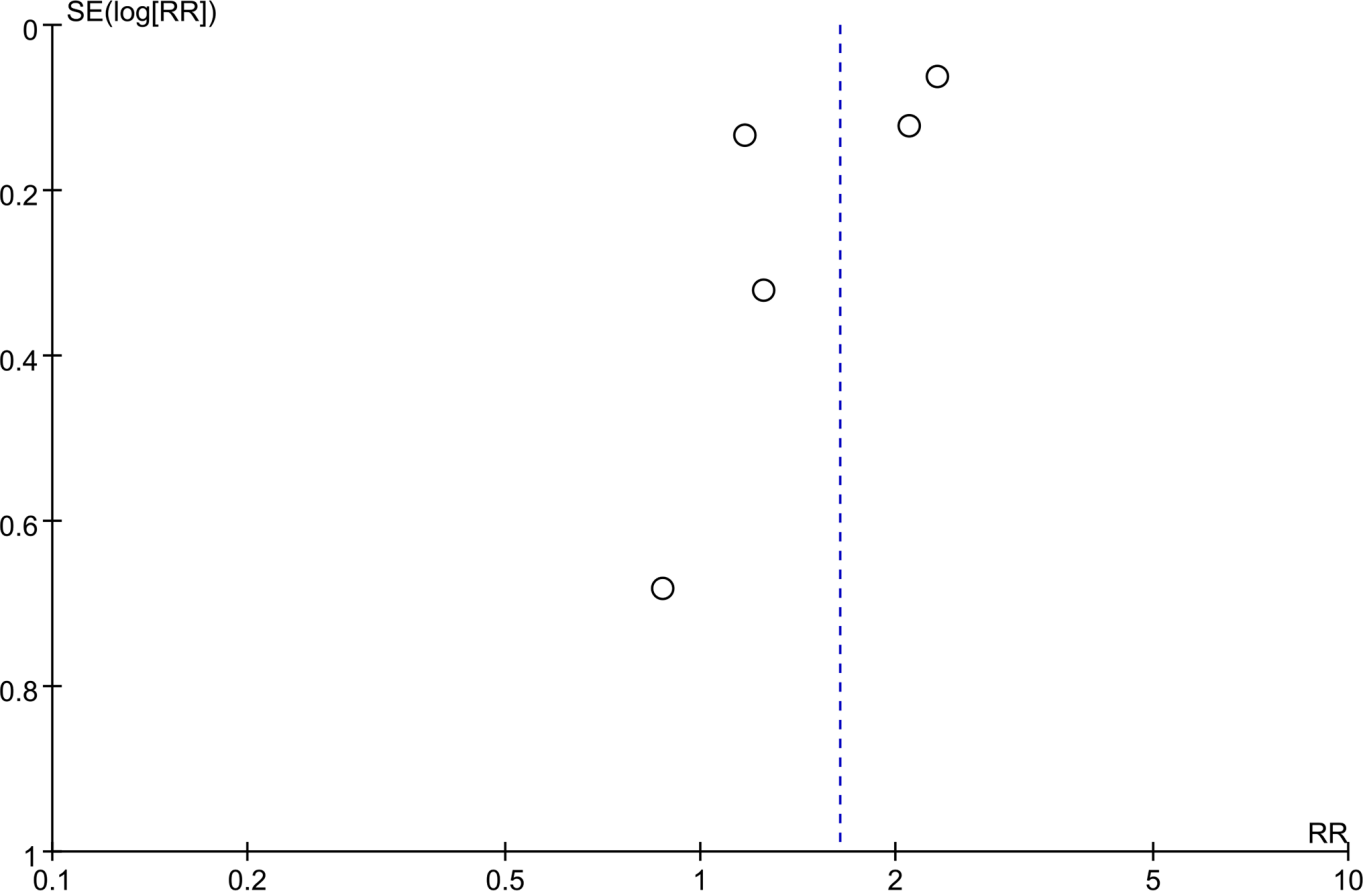
Funnel plots for all of the included studies reported in this meta-analysis.

The funnel plot did not indicate any evidence of publication bias (Fig 4).

## Discussion

At present, the relationship between PD-L1 and clinical outcomes in breast cancer patients remains unclear. In this study, we focused on the prognostic utility of PD-L1 in breast cancer and its relationship with the clinicopathological features of breast cancer patients. To our knowledge, this is the first meta-analysis to discuss this topic. From our analysis, we observed that the pooled RR value for mortality in breast cancer patients was 1.64 (95% CI, 1.14–2.34), which suggests that patients with positive/higher PD-L1 expression have significantly poorer outcomes, as compared to patients with negative/lower PD-L1 expression. To avoid bias caused by the follow-up duration after surgery, we assessed the MR_10years_ and found that the pooled RR of mortality 10 years after surgery was 2.53 (95% CI, 1.78–3.59), which is consistent with the abovementioned results.

Several antibodies that inhibit the PD-1 pathway (by blocking either PD-1 or PD-L1) are being developed for clinical use in various tumor types and clinical settings [22]. Researchers have observed that treatment with anti-PD-L1 antibody ibrutinib (a covalent inhibitor of Bruton’s tyrosine kinase) suppresses tumor growth in mouse models of triple-negative breast cancer (TNBC) [23]. Moreover, several clinical trials are ongoing wherein the PD-1/PD-L1 pathway is targeted with MEDI4736 or MPDL3280A, which are monoclonal antibodies against PD-L1. Notably, two early phase I trials involving PD-1/PD-L1 blockade (with pembrolizumab or MPDL3280A) in TNBC have demonstrated overall response rates of 15–20%[24, 25]. According to the preliminary report of a phase 1A trial (MPDL3280A) presented at the annual meeting of the American Association for Cancer Research’s in 2015, objective responses were noted in 24% of patients (95% CI 8–47); 10% patients showed complete responses and 14% patients showed partial responses; 29% patients had progression-free survival of 24 weeks or longer, after following up for 40 weeks[26]. Thus, treatment with PD-L1 could be an option in TNBC, cause all theses trails have been conducted in patients with TNBC, but not in other breast cancer subtypes. These findings suggest that the high expression of PD-L1 indicates a poor outcome and that treatment with anti-PD-L1 antibodies should be attempted in patients with breast carcinoma in future clinical trials.

While examining the relationship between PD-L1 expression and clinicopathological features, we observed that the pooled RR was significantly associated with lymph node metastasis, poor nuclear grade, and negative ER expression. These findings are consistent with the results of other studies, including the study by Mittendorf et al, wherein PD-L1+ carcinoma cells were observed more often in TNBC than in other breast cancer subtypes [6] and the study of Wimberly et al., wherein PD-L1 expression correlated with the lack of ER expression [27]. No previous studies have definitely indicated that nuclear grade and lymph node metastasis are related to PD-L1 expression in breast cancer, although some articles on other cancers have described these relationships for other cancer[18, 28, 29]. A large number of metastatic lymph nodes, poorer nuclear grade, and negative ER expression all indicate a poor outcome. These findings support the sensitivity and specificity of PD-L1 in the predicting clinical survival in patients with breast carcinoma. Thus, part of the patients with metastatic lymph nodes, poorer nuclear grade, and negative ER expression may benefit from anti-PD-L1 therapy, which would improve their prognosis and help improve the OS rate. However, wheter PD-L1 expression is a predictive marker for the response to anti-PD-L1 therapy in breast cancer patients is still a question need to be solved.

Three study were excluded from this meta-analysis because PD-L1 expression were detected using DNA microarray[30, 31] or fluorescent RNAscope paired-primer assay[32]. Clearly, comparing IHC-based protein expression with microarray-based gene-expression levels can lead to quite distinct conclusions. For example, DNA microarray-based measurements quantify expression levels in tumor cells, non-tumor cells, and infiltrating immune cells, while our present study focused on the expression of PD-L1 in tumor cells. Sabatier et al and Schalper et al concluded that PD-L1 upregulation was associated with better survival in patients with breast cancer especially basal-like breast cancer (express genes characteristic of the outer or basally located epithelial layer of the mammary gland)[31–33], while Bertucci et al considered PD-L1 overexpression in inflammatory breast cancer orrelated with better response to chemotherapy[30]. The differences in results between these studies may be mainly due to differences in the detection method used and the subtype of breast cancer.

Nevertheless, the present study has certain limitations. First, PD-L1 is a novel target that has not been extensively studied; in fact, most of the included studies had a relatively small sample size and could not be compared. Second, the antibodies used in each study were different, as were the sensitivities of the antibodies, the experimental procedures and immunohistochemical reagents, the scoring method, the cut-off value for PD-L1 overexpression, and the study end points varied. Collectively, these factors could lead to a high degree of heterogeneity. Third, potential factors such as the therapeutic strategy, patient age, and BMI, which were not considered in the meta-analysis may have impacted on our results, even if similar inclusion criteria were used for each study.

In conclusion, our findings revealed that PD-L1 should be considered as a prognostic indicator of poor survival in patients with breast cancer. Since high PD-L1 expression is associated to canonical prognostic factors of breast cancer, patients with positive PD-L1 expression may present with more extensive lymph node metastasis and poor nuclear grade. Even though PD-L1 expression does not add so much new insight for the prognostic characterization of the patient but it can be used for the selection of patients candidate to immunotherapy. Hence, in addition to endocrine therapy or chemotherapy, such patients may benefit from anti PD-L1 therapy. Furthermore, additional studies are needed from multiple centers with large sample sizes and detailed follow-up, in order to study the role of the PD-1/PD-L1 pathway in breast cancer.

## Supporting Information

S1 File Completed 2009 PRISMA Checklist. (DOC)

S2 File A full list of excluded articles and their reasons for exclusion. (DOC)
