# Supplementary material for: Prognostic and Clinicopathological Value of Programmed Death Ligand-1 in Breast Cancer: A Meta-Analysis
Source: PLoS One. 2016 May 26;11(5):e0156323. doi: 10.1371/journal.pone.0156323 (PMC4882023; doi:10.1371/journal.pone.0156323)
Supplement: S2 File — (DOC) [file pone.0156323.s002.doc]

**A full list of excluded articles and their reasons for exclusion**

1. duplicate publication[1-14];
2. non-human experiments[15-19]; non-English paper[20];
3. conference abstract[21-111]；
4. books, review articles, case reports, or letters[112-301];
5. not prognosis study about breast cancer[2, 3, 5, 7, 9, 11, 13, 302-355], insufficient data regarding 95% confidence interval (95% CI) and risk ratios (RR), or the Kaplan-Meier curve could not be extracted[1, 10, 14, 356-362].

**References**
